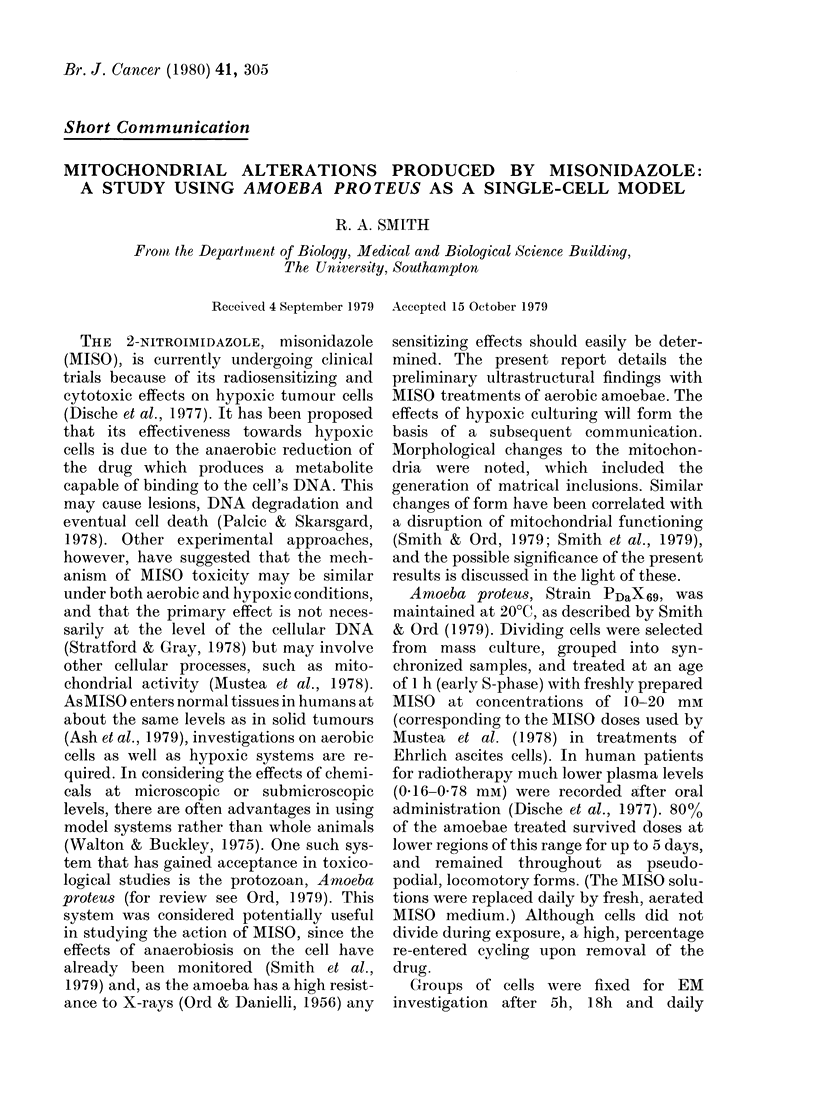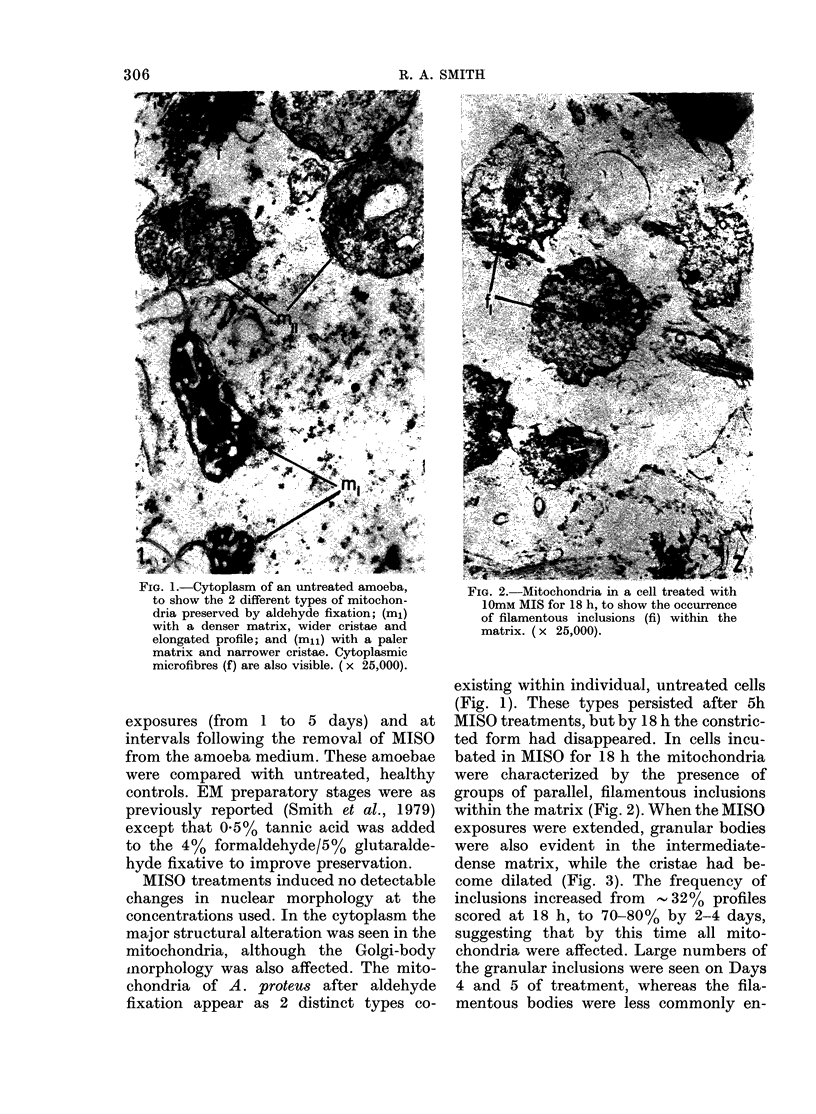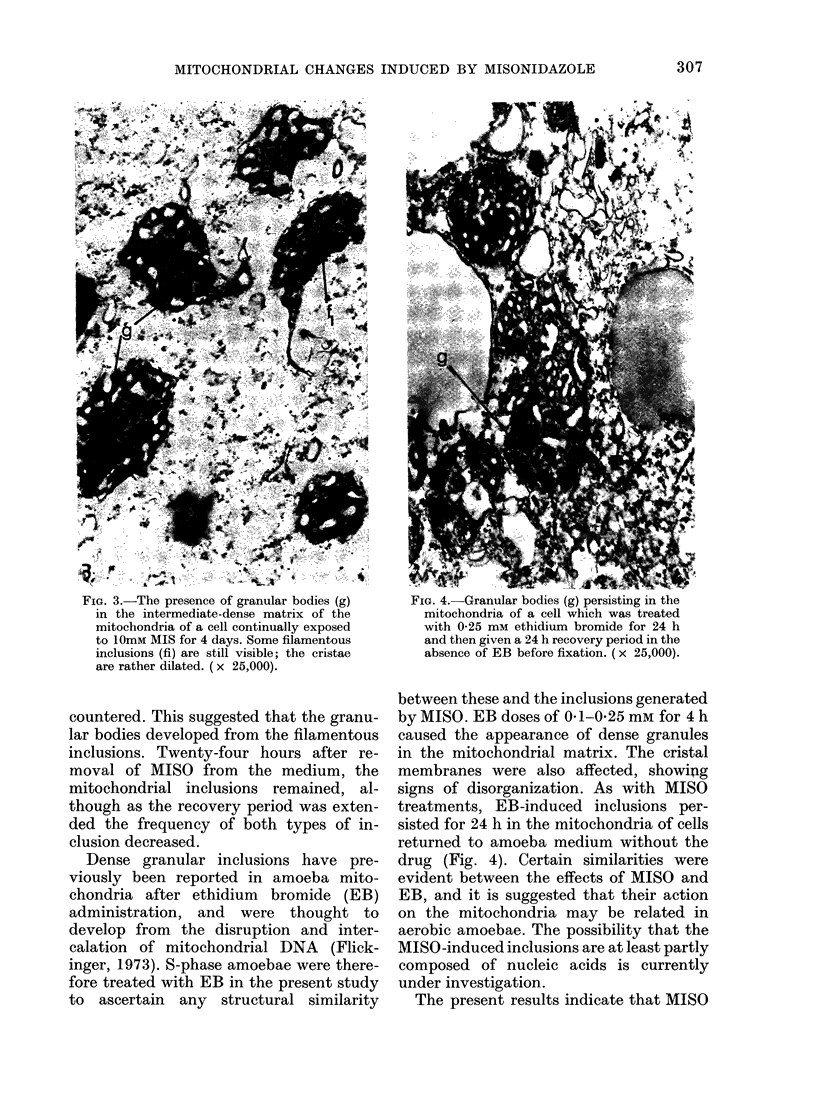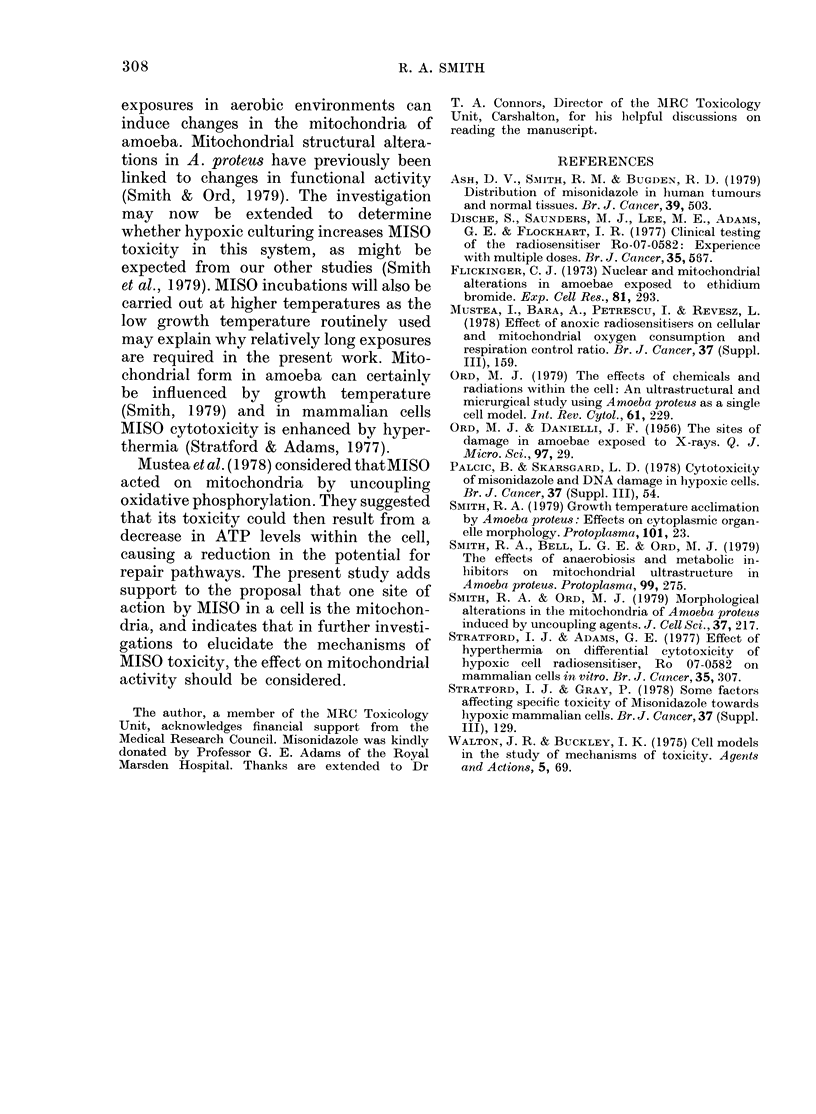# Mitochondrial alterations produced by misonidazole: a study using Amoeba proteus as a single-cell model.

**DOI:** 10.1038/bjc.1980.44

**Published:** 1980-02

**Authors:** R. A. Smith

## Abstract

**Images:**


*Br. J. Cancer* (1980) **41**, 305

***Short Communication***

### MITOCHONDRIAL ALTERATIONS PRODUCED BY MISONIDAZOLE: A STUDY USING *AMOEBA PROTEUS* AS A SINGLE-CELL MODEL

R. A. SMITH

*From the Department of Biology, Medical and Biological Science Building, The University, Southampton*

Received 4 September 1979  Accepted 15 October 1979

THE 2-NITROIMIDAZOLE, misonidazole (MISO), is currently undergoing clinical trials because of its radiosensitizing and cytotoxic effects on hypoxic tumour cells (Dische *et al.*, 1977). It has been proposed that its effectiveness towards hypoxic cells is due to the anaerobic reduction of the drug which produces a metabolite capable of binding to the cell's DNA. This may cause lesions, DNA degradation and eventual cell death (Palcic & Skarsgard, 1978). Other experimental approaches, however, have suggested that the mechanism of MISO toxicity may be similar under both aerobic and hypoxic conditions, and that the primary effect is not necessarily at the level of the cellular DNA (Stratford & Gray, 1978) but may involve other cellular processes, such as mitochondrial activity (Mustea *et al.*, 1978). As MISO enters normal tissues in humans at about the same levels as in solid tumours (Ash *et al.*, 1979), investigations on aerobic cells as well as hypoxic systems are required. In considering the effects of chemicals at microscopic or submicroscopic levels, there are often advantages in using model systems rather than whole animals (Walton & Buckley, 1975). One such system that has gained acceptance in toxicological studies is the protozoan, *Amoeba proteus* (for review see Ord, 1979). This system was considered potentially useful in studying the action of MISO, since the effects of anaerobiosis on the cell have already been monitored (Smith *et al.*, 1979) and, as the amoeba has a high resistance to X-rays (Ord & Danielli, 1956) any sensitizing effects should easily be determined. The present report details the preliminary ultrastructural findings with MISO treatments of aerobic amoebae. The effects of hypoxic culturing will form the basis of a subsequent communication. Morphological changes to the mitochondria were noted, which included the generation of matrical inclusions. Similar changes of form have been correlated with a disruption of mitochondrial functioning (Smith & Ord, 1979; Smith *et al.*, 1979), and the possible significance of the present results is discussed in the light of these.

*Amoeba proteus*, Strain $P_{Da}X_{69}$, was maintained at 20°C, as described by Smith & Ord (1979). Dividing cells were selected from mass culture, grouped into synchronized samples, and treated at an age of 1 h (early S-phase) with freshly prepared MISO at concentrations of 10–20 mM (corresponding to the MISO doses used by Mustea *et al.* (1978) in treatments of Ehrlich ascites cells). In human patients for radiotherapy much lower plasma levels (0·16–0·78 mM) were recorded after oral administration (Dische *et al.*, 1977). 80% of the amoebae treated survived doses at lower regions of this range for up to 5 days, and remained throughout as pseudopodial, locomotory forms. (The MISO solutions were replaced daily by fresh, aerated MISO medium.) Although cells did not divide during exposure, a high, percentage re-entered cycling upon removal of the drug.

Groups of cells were fixed for EM investigation after 5h, 18h and daily

FIG. 1.—Cytoplasm of an untreated amoeba, to show the 2 different types of mitochondria preserved by aldehyde fixation; ($m_{I}$) with a denser matrix, wider cristae and elongated profile; and ($m_{II}$) with a paler matrix and narrower cristae. Cytoplasmic microfibres (f) are also visible. (× 25,000).

FIG. 2.—Mitochondria in a cell treated with 10mM MIS for 18 h, to show the occurrence of filamentous inclusions (fi) within the matrix. (× 25,000).

exposures (from 1 to 5 days) and at intervals following the removal of MISO from the amoeba medium. These amoebae were compared with untreated, healthy controls. EM preparatory stages were as previously reported (Smith *et al.*, 1979) except that 0·5% tannic acid was added to the 4% formaldehyde/5% glutaraldehyde fixative to improve preservation.

MISO treatments induced no detectable changes in nuclear morphology at the concentrations used. In the cytoplasm the major structural alteration was seen in the mitochondria, although the Golgi-body morphology was also affected. The mitochondria of *A. proteus* after aldehyde fixation appear as 2 distinct types coexisting within individual, untreated cells (Fig. 1). These types persisted after 5h MISO treatments, but by 18 h the constricted form had disappeared. In cells incubated in MISO for 18 h the mitochondria were characterized by the presence of groups of parallel, filamentous inclusions within the matrix (Fig. 2). When the MISO exposures were extended, granular bodies were also evident in the intermediate-dense matrix, while the cristae had become dilated (Fig. 3). The frequency of inclusions increased from ~32% profiles scored at 18 h, to 70–80% by 2–4 days, suggesting that by this time all mitochondria were affected. Large numbers of the granular inclusions were seen on Days 4 and 5 of treatment, whereas the filamentous bodies were less commonly en-

FIG. 3.—The presence of granular bodies (g) in the intermediate-dense matrix of the mitochondria of a cell continually exposed to 10mM MIS for 4 days. Some filamentous inclusions (fi) are still visible; the cristae are rather dilated. (× 25,000).

FIG. 4.—Granular bodies (g) persisting in the mitochondria of a cell which was treated with 0·25 mM ethidium bromide for 24 h and then given a 24 h recovery period in the absence of EB before fixation. (× 25,000).

countered. This suggested that the granular bodies developed from the filamentous inclusions. Twenty-four hours after removal of MISO from the medium, the mitochondrial inclusions remained, although as the recovery period was extended the frequency of both types of inclusion decreased.

Dense granular inclusions have previously been reported in amoeba mitochondria after ethidium bromide (EB) administration, and were thought to develop from the disruption and intercalation of mitochondrial DNA (Flickinger, 1973). S-phase amoebae were therefore treated with EB in the present study to ascertain any structural similarity between these and the inclusions generated by MISO. EB doses of 0·1–0·25 mM for 4 h caused the appearance of dense granules in the mitochondrial matrix. The cristal membranes were also affected, showing signs of disorganization. As with MISO treatments, EB-induced inclusions persisted for 24 h in the mitochondria of cells returned to amoeba medium without the drug (Fig. 4). Certain similarities were evident between the effects of MISO and EB, and it is suggested that their action on the mitochondria may be related in aerobic amoebae. The possibility that the MISO-induced inclusions are at least partly composed of nucleic acids is currently under investigation.

The present results indicate that MISO

exposures in aerobic environments can induce changes in the mitochondria of amoeba. Mitochondrial structural alterations in *A. proteus* have previously been linked to changes in functional activity (Smith & Ord, 1979). The investigation may now be extended to determine whether hypoxic culturing increases MISO toxicity in this system, as might be expected from our other studies (Smith *et al.*, 1979). MISO incubations will also be carried out at higher temperatures as the low growth temperature routinely used may explain why relatively long exposures are required in the present work. Mitochondrial form in amoeba can certainly be influenced by growth temperature (Smith, 1979) and in mammalian cells MISO cytotoxicity is enhanced by hyperthermia (Stratford & Adams, 1977).

Mustea *et al.* (1978) considered that MISO acted on mitochondria by uncoupling oxidative phosphorylation. They suggested that its toxicity could then result from a decrease in ATP levels within the cell, causing a reduction in the potential for repair pathways. The present study adds support to the proposal that one site of action by MISO in a cell is the mitochondria, and indicates that in further investigations to elucidate the mechanisms of MISO toxicity, the effect on mitochondrial activity should be considered.

The author, a member of the MRC Toxicology Unit, acknowledges financial support from the Medical Research Council. Misonidazole was kindly donated by Professor G. E. Adams of the Royal Marsden Hospital. Thanks are extended to Dr T. A. Connors, Director of the MRC Toxicology Unit, Carshalton, for his helpful discussions on reading the manuscript.

### REFERENCES

Ash, D. V., Smith, R. M. & Bugden, R. D. (1979) Distribution of misonidazole in human tumours and normal tissues. *Br. J. Cancer*, **39**, 503.

Dische, S., Saunders, M. J., Lee, M. E., Adams, G. E. & Flockhart, I. R. (1977) Clinical testing of the radiosensitiser Ro-07-0582: Experience with multiple doses. *Br. J. Cancer*, **35**, 567.

Flickinger, C. J. (1973) Nuclear and mitochondrial alterations in amoebae exposed to ethidium bromide. *Exp. Cell Res.*, **81**, 293.

Mustea, I., Bara, A., Petrescu, I. & Revesz, L. (1978) Effect of anoxic radiosensitisers on cellular and mitochondrial oxygen consumption and respiration control ratio. *Br. J. Cancer*, **37** (Suppl. III), 159.

Ord, M. J. (1979) The effects of chemicals and radiations within the cell: An ultrastructural and micrurgical study using *Amoeba proteus* as a single cell model. *Int. Rev. Cytol.*, **61**, 229.

Ord, M. J. & Danielli, J. F. (1956) The sites of damage in amoebae exposed to X-rays. *Q. J. Micro. Sci.*, **97**, 29.

Palcic, B. & Skarsgard, L. D. (1978) Cytotoxicity of misonidazole and DNA damage in hypoxic cells. *Br. J. Cancer*, **37** (Suppl. III), 54.

Smith, R. A. (1979) Growth temperature acclimation by *Amoeba proteus*: Effects on cytoplasmic organelle morphology. *Protoplasma*, **101**, 23.

Smith, R. A., Bell, L. G. E. & Ord, M. J. (1979) The effects of anaerobiosis and metabolic inhibitors on mitochondrial ultrastructure in *Amoeba proteus*. *Protoplasma*, **99**, 275.

Smith, R. A. & Ord, M. J. (1979) Morphological alterations in the mitochondria of *Amoeba proteus* induced by uncoupling agents. *J. Cell Sci.*, **37**, 217.

Stratford, I. J. & Adams, G. E. (1977) Effect of hyperthermia on differential cytotoxicity of hypoxic cell radiosensitiser, Ro 07-0582 on mammalian cells *in vitro*. *Br. J. Cancer*, **35**, 307.

Stratford, I. J. & Gray, P. (1978) Some factors affecting specific toxicity of Misonidazole towards hypoxic mammalian cells. *Br. J. Cancer*, **37** (Suppl. III), 129.

Walton, J. R. & Buckley, I. K. (1975) Cell models in the study of mechanisms of toxicity. *Agents and Actions*, **5**, 69.